# Immunogenicity and safety of an *Escherichia coli*-produced bivalent human papillomavirus vaccine (Cecolin) in girls aged 9–14 years in Ghana and Bangladesh: a randomised, controlled, open-label, non-inferiority, phase 3 trial

**DOI:** 10.1016/S1473-3099(25)00031-3

**Published:** 2025-08

**Authors:** Tsiri Agbenyega, Anne E Schuind, Samuel Adjei, Kalpana Antony, John J Aponte, Patrick B Y Buabeng, John D Clemens, Lokman Hossain, Troy J Kemp, Laina D Mercer, Ligia A Pinto, Firdausi Qadri, Kristen Sukraw, Niranjan Bhat, Khalequ Zaman

**Affiliations:** a Malaria Research Center, Agogo Presbyterian Hospital/Kwame Nkrumah University of Science and Technology, Agogo, Ghana; b PATH, Center for Vaccine Innovation and Access, Seattle, WA, USA; c International Vaccine Institute, Seoul, South Korea; d Center for Vaccine Innovation, Seoul, South Korea; e UCLA Fielding School of Public Health, Los Angeles, CA, USA; f Infectious Diseases Division, International Centre for Diarrhoeal Disease Research, Dhaka, Bangladesh; g HPV Serology Laboratory, Vaccine, Immunity, and Cancer Directorate, Frederick National Laboratory for Cancer Research, Frederick, MD, USA

## Abstract

**Background:**

Human papillomavirus (HPV) vaccines have been available for nearly 20 years. However, the overall coverage of girls aged 15 years and younger is low, especially in low-resource settings, where the burden of cervical cancer is highest. Increasing access and facilitating implementation of HPV vaccination will contribute to cervical cancer elimination efforts. To generate data in different dosing regimens and in low-resource settings, we aimed to evaluate the safety and immunogenicity of various schedules of an *Escherichia coli*-expressed bivalent HPV vaccine (2vHPV) compared with a widely used quadrivalent vaccine.

**Methods:**

This randomised, controlled, open-label, non-inferiority, phase 3 trial enrolled healthy girls aged 9–14 years from single sites in Ghana and Bangladesh. Participants were randomly assigned via interactive web response system technology equally into five study groups, stratified by site: two doses of 2vHPV, the first at baseline and the second 6, 12, or 24 months later; a quadrivalent HPV vaccine (4vHPV) at baseline followed 24 months later by 2vHPV; or two doses of 4vHPV given 6 months apart (referent). We tested for antigen-specific (HPV-16 and HPV-18) binding antibodies by ELISA at baseline and before and 1 month after the second dose. The primary objective was to show immunological non-inferiority of the 2vHPV vaccine schedules to the referent 1 month after the second dose in the per-protocol population, with a non-inferiority margin of 0·5 for the lower bound of the 98·3% CI for the geometric mean concentration (GMC) ratio. Adverse events and serious adverse events were evaluated as secondary endpoints in the total vaccinated population. The study is registered at ClinicalTrials.gov (NCT04508309) and is completed.

**Findings:**

Between March 15 and Nov 18, 2021, 1025 girls were enrolled and received 2vHPV at baseline and 6 months (n=205), 12 months (n=206), or 24 months (n=204); 4vHPV at baseline and 6 months (n=205); or 4vHPV at baseline and 2vHPV at 24 months (n=205). 96–99% of participants across groups were included in the per-protocol analysis. 1 month after the second dose, 2vHPV non-inferiority was shown, with GMC ratios between 1·1 and 2·4 (lower bound of the 98·3% CI of the GMC ratio between 0·9 and 1·9) for HPV-16, and between 1·3 and 1·7 (1·0 and 1·4) for HPV-18. As an exploratory objective, we assessed 2vHPV immunogenicity after one dose, finding that it was similar to that of 4vHPV up to 24 months, with GMC ratios at 24 months of 1·1 (95% CI 0·9–1·4) for HPV-16 and 1·4 (1·1–1·7) for HPV-18. The frequency of adverse events was similar across study groups, with no related unsolicited events reported. Serious adverse events were rare and none were determined to be related to vaccination.

**Interpretation:**

Non-inferior immune responses for extended two-dose regimens of 2vHPV support dosing flexibility. For up to 24 months, one dose of 2vHPV elicited immunogenicity that was similar to one dose of 4vHPV, for which single-dose efficacy has been shown, supporting a single-dose use of 2vHPV.

**Funding:**

The Bill & Melinda Gates Foundation and the German Federal Ministry of Education and Research and immunological testing was funded in part by the National Cancer Institute, National Institutes of Health.

**Translation:**

For the Bengali translation of the abstract see Supplementary Materials section.

## Introduction

Persistent infections with human papillomavirus (HPV) types 16 and 18 are responsible for the majority of cervical cancers and HPV-related cancers at other anatomical sites. Worldwide, cervical cancer represents the highest burden of HPV-associated cancers, with 660 000 new cases and 350 000 deaths in 2022, 90% of which occurred in low-income and middle-income countries (LMICs).^1^ HPV infections are preventable with HPV vaccination before exposure.^2^ Prophylactic HPV vaccines were first introduced in 2006; yet to date, only 20% of girls younger than 15 years (the primary target population for vaccination) have been vaccinated.^3^ Based on growing evidence that a one-dose regimen is as efficacious as two or three doses, WHO updated their recommendation in 2022 to include a single-dose regimen as an acceptable off-label alternative to the two-dose regimen in girls and women aged 9–20 years.^4^

**Research in context**

**Evidence before this study**

We searched PubMed for clinical trials published between database inception and Dec 8, 2024, using the MeSH terms “papillomavirus vaccines”, “antibodies, viral”, “child” or “adolescent”, and “human”, and publication type “randomized control trial”, with no language restrictions, and hand-searched the reference lists from the resulting publications. We identified 69 trials, of which 21 evaluated MSD's quadrivalent human papillomavirus (HPV) vaccine (4vHPV; Gardasil), nine evaluated MSD's nonavalent vaccine (Gardasil 9), 20 evaluated GSK's bivalent vaccine (Cervarix), eight evaluated combinations of these three, five evaluated Innovax's bivalent HPV vaccine (2vHPV; Cecolin), three evaluated Innovax's nonavalent vaccine (in clinical development), and three evaluated Zerun/Walvax's bivalent vaccine (Walrinvax). Except for one publication related to the interim analysis of our study, all other trials of Innovax's bivalent vaccine were conducted in China and most were in adults. One study of Innovax's bivalent vaccine included a population of girls aged 9–17 years and described single-dose immunogenicity up to 6 months, in addition to the primary outcomes related to immunobridging of a two-dose regimen (6 months apart) or a three-dose regimen (baseline, 1 month, and 6 months) in girls aged 9–17 years to an adult population. The only publication to evaluate Innovax's bivalent vaccine in alternate two-dose schedules or single-dose immunogenicity compared with a licensed HPV vaccine was the interim analysis of this study, which included data up to 7 months for the two groups that received vaccines at baseline and after 6 months. Innovax's bivalent HPV vaccine had also not been previously compared with another licensed HPV vaccine, nor had vaccine interchangeability with another HPV vaccine been assessed.

**Added value of this study**

Our research compared extended two-dose schedules and a mixed-dose schedule of 2vHPV to 4vHPV in girls aged 9–14 years in Ghana and Bangladesh to generate data with a broader geographical representation and in low-resource settings. Further, the study design allowed for an evaluation of single-dose immunogenicity of 2vHPV up to 24 months, supporting immunobridging. Immunological testing was performed with product-agnostic antigens in the serology assays.

**Implications of all the available evidence**

This evidence provides additional data supporting the safety and effectiveness of 2vHPV in low-income settings. Since 2vHPV is WHO prequalified, this vaccine represents an affordable option and increases the overall supply of HPV vaccines in low-income and middle-income countries. The satisfactory immunogenicity of the extended two-dose regimens of 2vHPV and the mixed-vaccine schedule supports flexibility in dosing and vaccine interchangeability. These data will facilitate immunisation implementation and mitigate the effect of external factors disrupting vaccine delivery, such as public health challenges or supply constraints. Finally, in the context of an increasing number of countries adopting a single-dose schedule, the effectiveness of one dose of 2vHPV can be inferred based on the demonstration of similar immunogenicity to 4vHPV for HPV-16 and HPV-18, the two most common oncogenic HPV types. Our data supported the decision by WHO to include 2vHPV in the list of vaccines recommended for single-dose use.

There are currently six licensed HPV vaccines, five of which have been prequalified by WHO, a prerequisite for procurement by UNICEF to countries eligible for Gavi, the Vaccine Alliance support.^5^ However, there are financial and programmatic difficulties for incorporating HPV vaccination into immunisation programmes. Therefore, an increased supply of affordable HPV vaccines is crucial for initial adoption and to mitigate any potential supply disruptions.

Innovax's bivalent HPV-16 and HPV-18 vaccine (2vHPV; Cecolin) obtained licensure in 2019 in China based on efficacy data showing prevention of high-grade cervical lesions in an adult population^6^ and immunological non-inferiority in Chinese adolescents compared with an adult population in which efficacy was shown.^7^ 2vHPV obtained WHO prequalification in 2021, and as of August, 2024, it has been licensed in 19 countries worldwide.^8^

This trial aimed to evaluate the safety and immunogenicity of 2vHPV given in different dosing regimens, compared with a widely used quadrivalent HPV vaccine (HPV-6, HPV-11, HPV-16, and HPV-18; 4vHPV; Gardasil), in girls from Ghana and Bangladesh aged 9–14 years. The study aimed to evaluate extended two-dose schedules, vaccine interchangeability, and the immunogenicity of a single dose of 2vHPV up to 24 months. An interim analysis was published in March, 2024.^9^ This Article reports on the final study data.

## Methods

### Study design

This randomised, active-comparator controlled, open-label, non-inferiority, phase 3 trial was performed at a single site in Ghana (the Malaria Research Center) and at a single site in Bangladesh (the International Centre for Diarrhoeal Disease Research). 4vHPV was selected as the active comparator because it is the most widely WHO-prequalified HPV vaccine used in LMICs, is licensed in both study countries, and has been shown to be efficacious in a single‑dose regimen,^10^ making it an appropriate reference for the study, including for immunobridging. The trial was registered before enrolment began in ClinicalTrials.gov (NCT04508309) and the Pan African Clinical Trials Registry (PACTR202008675647876). The study protocol is available online. A protocol safety review team composed of the site principal investigators, the sponsor, and the contract research organisation medical monitors routinely monitored safety throughout the trial.

This study was conducted in accordance with the ethical principles set forth in the World Medical Association Declaration of Helsinki and the Council for International Organizations of Medical Sciences International Ethical Guidelines for Biomedical Research Involving Human Participants and in conformity with Good Clinical Practice and local regulatory requirements. The study was approved by the WIRB-Copernicus Group institutional review board and by ethics committees and regulatory authorities of both countries.

### Participants

Participants were recruited through community outreach. Girls aged 9–14 years were eligible for the study if they were healthy (as determined by medical history and physical examination by a clinician), were living in reasonable proximity to the study site, and did not have plans to leave the area for 25 months. Key exclusion criteria included a history of HPV vaccination, allergies to components of the study vaccines, current pregnancy or breastfeeding, history of immunocompromising conditions (including HIV infection), and presence of an acute disease on the day of vaccination. The full list of eligibility criteria can be found on ClinicalTrials.gov.

Written informed consent from parents or legally acceptable representatives and, as applicable, assent from study participants were obtained before study participation. The principal investigators ensured that parents and participants were fully informed about the aims, procedures, potential risks, and potential benefits of the study. Participation in the study was strictly voluntary.

### Randomisation and masking

Participants were randomly assigned in blocks of five in a 1:1:1:1:1 ratio to one of the study groups: two doses of 2vHPV given 6, 12, or 24 months apart; one dose of 4vHPV followed 24 months later by a single dose of 2vHPV (mixed-dose schedule); or two doses of 4vHPV 6 months apart (referent). Randomisation was stratified by study site and done via an interactive web response system based on a list prepared by a separate team who had no further involvement in enrolment. Through the same system, approximately 20% of participants from each group were randomly selected to also participate in pseudovirion-based neutralisation assay (PBNA) testing. The study was open label; however, the individuals who performed serology testing were masked to group assignment and did not receive the participant group assignments.

### Procedures

After informed consent and assent (as appropriate) were obtained, participants were screened for eligibility, including a medical history with clinical examination and, in girls of childbearing potential, a urine pregnancy test. Within 28 days of the screening visit, eligible participants were randomly assigned. Between six and eight study visits were scheduled during the study period, depending on whether screening was performed before or on study day 1 and depending on the study group. Windows for study visits were predefined in the protocol. Following a negative pregnancy test, study vaccines were administered intramuscularly into the deltoid muscle. 2vHPV is manufactured by Xiamen Innovax Biotech in Xiamen, China. Each dose contains L1 capsid proteins of HPV-16 (40 μg) and HPV-18 (20 μg) adsorbed on aluminium hydroxide adjuvant. L1 antigens, expressed in *Escherichia coli* by recombinant technology, self-assemble into virus‑like particles. Each comparator dose of 4vHPV (manufactured by MSD, Haarlem, the Netherlands) contains L1 capsid proteins of HPV-6 (20 μg), HPV-11 (40 μg), HPV-16 (40 μg), and HPV-18 (20 μg) adsorbed on amorphous aluminium hydroxyphosphate sulphate adjuvant. HPV antigens are expressed in *Saccharomyces cerevisiae* by recombinant technology, each self-assembling into virus‑like particles.

**Figure 1:**
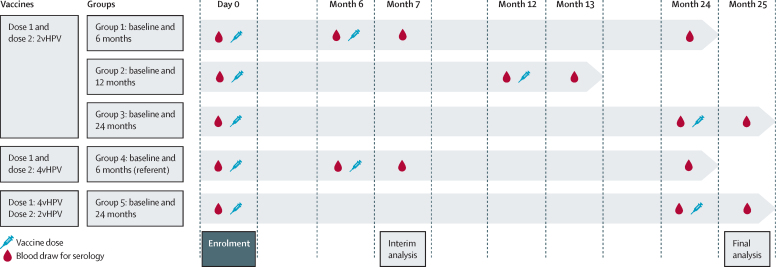
Study design HPV=human papillomavirus. 2vHPV=bivalent HPV vaccine. 4vHPV=quadrivalent HPV vaccine.

Participants visited the study site for safety assessment and collection of a memory aid 1 week and 1 month after vaccination. Serum was obtained at baseline and before and 1 month after the second dose. Additional blood collection occurred 18 months after the second dose in the two study groups on a 6‑month schedule (figure 1).

Safety was assessed as previously described.^9^ Briefly, the study staff monitored participants for solicited reactions within 30 min of vaccination. Participants or their legal representatives used a memory aid to report solicited adverse events daily for 7 days and other adverse events for 30 days post-vaccination. Adverse events were graded from mild (grade 1) to life-threatening (grade 4). Grade 5 was assigned to any adverse event leading to death. Relatedness was attributed by study investigators if there was a reasonable possibility of causality to study vaccines. Adverse events were classified according to the Medical Dictionary for Regulatory Activities.

Immunological testing was performed by the Frederick National Laboratory for Cancer Research in the USA. It included measurement of antigen-specific (HPV-16 or HPV-18) binding antibodies (ELISA) as the primary assay, and in a random sample of participants, neutralising antibodies by PBNA. Samples from all study groups were tested within the same batch to minimise variability and increase reliability of comparisons between vaccines. The HPV-16 and HPV-18 virus‑like particles and pseudovirion particles used as assay antigens were produced in a mammalian cell system, independent of the vaccine manufacturer's production system. Both assays are described in appendix 2 (p 2). The lower limit of quantitation for the HPV-16 ELISA assay was 1·4 international unit (IU)/mL, and it was 1·1 IU/mL for the HPV-18 ELISA assay. The lower limit of quantitation for the HPV-16 PBNA was a titre of less than 21, and it was less than 16 for the HPV-18 PBNA.

### Outcomes

The coprimary study objectives, based on HPV-16 and HPV-18 IgG antibody levels, were the demonstration of immunological non-inferiority of 2vHPV 1 month after the second dose of the two-dose regimens given 6 months, 12 months, or 24 months apart, compared with two doses of 4vHPV given 6 months apart. The statistical analysis was performed on all data (safety and immunological) from all sites by an external statistician. Secondary objectives were neutralising antibody responses and binding antibody seroconversion rates (four-fold increase of antibody levels from baseline); non-inferiority based on antibody concentrations 1 month after the second dose of the mixed-dose schedule compared with 1 month after the second dose of the 4vHPV referent regimen; and non-inferiority based on antibody concentrations of 2vHPV to 4vHPV 18 months after the second dose when both were given as two doses, 6 months apart. We also evaluated safety as a secondary objective, with the outcomes of solicited adverse events within 30 min and 7 days, unsolicited adverse events within 1 month after each dose, and serious adverse events occurring at any time throughout the study. A key prespecified exploratory objective was to evaluate the persistence of the immune response by binding (ELISA) and neutralising (PBNA) antibodies after one dose of 2vHPV or 4vHPV, based on samples collected before second dose administration.

### Statistical analysis

We performed an interim analysis of both study groups on a baseline and 6-month schedule up to month 7.^9^ Non-inferiority for each of the three co-primary objectives was declared when the lower bound of the 98·3% CI of the geometric mean concentration (GMC) ratio was greater than 0·5. This analysis required a sample size of 205 participants per group to provide 90% power for the simultaneous assessment of non-inferiority for serotypes HPV-16 and HPV-18, assuming an antibody SD of 0·65 on the log_10_ scale,^11^ 10% baseline seropositivity,^7^, ^12^ 15% loss to follow-up,^12^ and a one-sided α of 0·0083, with Bonferroni correction. For secondary objectives, non-inferiority was declared when the lower bound of the 95% CI of the GMC ratio was greater than 0·5.

GMCs, geometric mean titres (GMTs), and GMC and GMT ratios, with corresponding CIs, were calculated using a linear model, with the log-transformed concentrations or titres as the dependent variable, study group as the explanatory variable, and study site as a covariate. Concentrations below the limit of quantification were considered censored, and a regression method that incorporated censoring was used to estimate the GMCs. We back-transformed log-scale coefficients to compute the estimates and corresponding CIs on their original scale. All GMC ratios were computed with the 2vHPV groups as the numerator. All participant-level percentages were supplemented with two-sided 95% CIs computed via the Clopper-Pearson method. Correlation between PBNA titres and IgG antibody concentrations was assessed via the Spearman correlation coefficient on the continuous scale. All analyses were performed in SAS version 9.4.

The primary and secondary immunogenicity analyses were conducted in the per-protocol population—participants with no major protocol deviations that would affect immune response and who were seronegative at baseline for the relevant HPV serotype—and included participants' data up to the time of a disqualifying event. We reviewed deviations and made exclusion decisions before analysis. All safety assessments took place in the total vaccinated population (those who received at least one dose of study vaccine), according to the treatment received. An independent multi-disciplinary data and safety monitoring board provided additional study oversight.

### Role of the funding source

The Bill & Melinda Gates Foundation provided initial recommendations regarding study design but had no role in protocol finalisation, data collection, data analysis, data interpretation, writing of the report, or the decision to submit for publication. The German Federal Ministry of Education and Research and the National Cancer Institute had no role in any of these activities.

## Results

**Figure 2:**
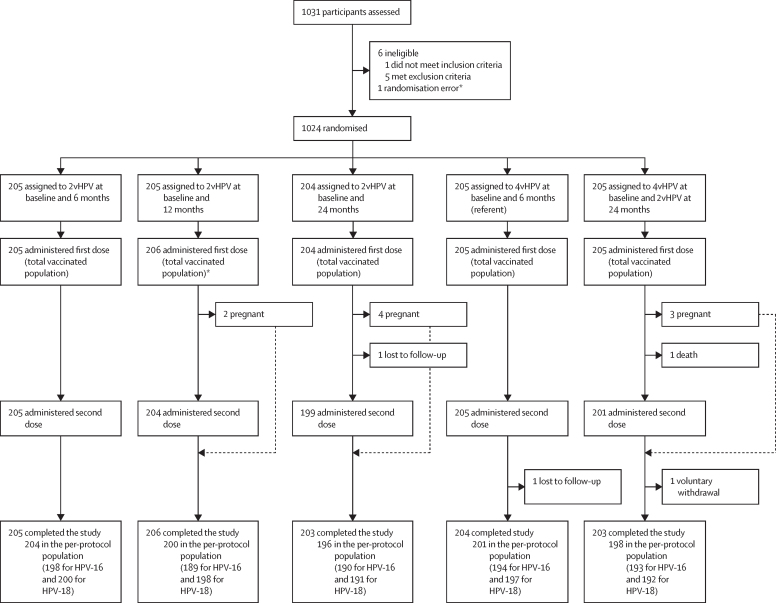
Trial profile Reasons for exclusion from the per-protocol population are provided in detail in appendix 2 (p 3) but include baseline seropositivity, missing results for a given timepoint, missed second dose, randomisation error, met exclusion criteria, early termination, and missed visit or sample taken outside of window. Participants could be excluded for multiple reasons. HPV=human papillomavirus. 2vHPV=bivalent HPV vaccine. 4vHPV=quadrivalent HPV vaccine. *Due to a site error, after eligibility was confirmed but before randomisation, a participant received the vaccine intended for a different participant. This single participant was included in the study for longitudinal evaluation but was excluded from the per-protocol analysis.

1031 girls aged 9–14 years were screened between March 15 and Nov 4, 2021; of them, six did not satisfy eligibility criteria. A total of 1025 girls (675 participants at the site in Bangladesh and 350 at the site in Ghana) were enrolled in the study. Between March 15, 2021, and Nov 18, 2021, approximately 205 participants were randomly assigned to each of the study groups (figure 2). All enrolled participants received the first vaccination and 1014 (99%) of 1025 participants received the second vaccination. 1021 participants completed follow-up; reasons for non-completion were death (one participant), loss to follow-up (two participants), and withdrawal of consent (one participant; figure 2).

In the total vaccinated population, 675 (66%) of 1025 participants were Asian and 350 (34%) of 1025 participants were Black. The participants' mean age was 11·3 years (SD 1·4). The distribution by race, age, weight, and height was similar between the study groups (table 1). At baseline by ELISA, 45 (4%) of 1025 participants were seropositive for HPV-16 and 31 (3%) of 1025 participants were seropositive for HPV-18. In the immunogenicity subset by PBNA, three (2%) of 205 participants were seropositive for HPV-16 and two (1%) of 205 participants were seropositive for HPV-18. The per-protocol population comprised 964 (94%) of 1025 participants for HPV-16 and 978 (95%) of 1025 participants for HPV-18 immunogenicity evaluation by ELISA (figure 2); and 199 (97%) of 205 participants for HPV-16 and 200 (98%) of 205 participants for HPV-18 evaluation by PBNA.

**Table 1:** Demographics of study participants at baseline (total vaccinated cohort)

		**2vHPV at baseline and 6 months (n=205)**	**2vHPV at baseline and 12 months (n=206)**	**2vHPV at baseline and 24 months (n=204)**	**4vHPV at baseline and 6 months (n=205)**	**4vHPV at baseline and 2vHPV at 24 months (n=205)**	**All participants (n=1025)**
Race							
	Asian	135 (66%)	136 (66%)	134 (66%)	135 (66%)	135 (66%)	675 (66%)
	Black	70 (34%)	70 (34%)	70 (34%)	70 (34%)	70 (34%)	350 (34%)
Age, years		11·4 (1·5)	11·4 (1·4)	11·4 (1·5)	11·3 (1·4)	11·3 (1·5)	11·3 (1·4)
Weight, kg		37·2 (10·4)	36·8 (9·5)	37·6 (10·9)	36·9 (9·5)	36·4 (10·7)	37·0 (10·2)
Height, cm		145·5 (10·4)	145·3 (9·5)	144·2 (10·3)	145·1 (9·3)	143·9 (10·2)	144·8 (10·0)

Data are n (%) or mean (SD). HPV=human papillomavirus. 2vHPV=bivalent HPV vaccine. 4vHPV=quadrivalent HPV vaccine.

2vHPV showed non-inferiority to the referent 1 month after the second dose for both antigens in all schedules, with IgG GMC ratios between 1·1 and 2·4 for HPV-16 and between 1·3 and 1·7 for HPV-18. For all three comparisons, the 98·3% CI lower bounds for the GMC ratios were above 0·5, the predefined non-inferiority margin, with values between 0·9 and 1·9 for HPV-16 and between 1·0 and 1·4 for HPV-18 (table 2; appendix 2 p 4).

**Table 2:** HPV-16 and HPV-18 binding (ELISA) antibody responses (per-protocol population)

		**2vHPV at baseline and 6 months (N=205)**	**2vHPV at baseline and 12 months (N=206)**	**2vHPV at baseline and 24 months (N=204)**	**4vHPV at baseline and 6 months (N=205)**	**4vHPV at baseline and 2vHPV at 24 months (N=205)**
**6 months after first dose**						
HPV-16						
	n	198	NA	NA	196	NA
	GMC (95% CI)	18 (16–20)	NA	NA	11 (10–12)	NA
	Seropositivity rate (95% CI)	100% (98·2–100)	NA	NA	99·5% (97·2–100)	NA
	GMC ratio to referent (95% CI)	1·5 (1·3–1·8)	NA	NA	Ref	NA
HPV-18						
	n	200	NA	NA	199	NA
	GMC (95% CI)	7 (6–7)	NA	NA	4 (4–5)	NA
	Seropositivity rate (95% CI)	98·5% (95·7–99·7)	NA	NA	96·0% (92·2–98·2)	NA
	GMC ratio to referent (95% CI)	1·6 (1·4–1·9)	NA	NA	Ref	NA
**12 months after first dose**						
HPV-16						
	n	NA	191	NA	NA	NA
	GMC (95% CI)	NA	13 (11–15)	NA	NA	NA
	Seropositivity rate (95% CI)	NA	99·5% (97·1–100)	NA	NA	NA
	GMC ratio to referent (95% CI)	NA	NA	NA	NA	NA
HPV-18						
	n	NA	200	NA	NA	NA
	GMC (95% CI)	NA	4 (4–5)	NA	NA	NA
	Seropositivity rate (95% CI)	NA	95·5% (91·6–97·9)	NA	NA	NA
	GMC ratio to referent (95% CI)	NA	NA	NA	NA	NA
**24 months after first dose**						
HPV-16						
	n	NA	NA	193	NA	197
	GMC (95% CI)	NA	NA	12 (10–14)	NA	11 (9–12)
	Seropositivity rate (95% CI)	NA	NA	97·9% (94·8–99·4)	NA	99·0% (96·4–99·9)
	GMC ratio to referent (95% CI)	NA	NA	1·1 (0·9–1·4)	NA	Ref
HPV-18						
	n	NA	NA	195	NA	196
	GMC (95% CI)	NA	NA	4 (4–5)	NA	3 (3–4)
	Seropositivity rate (95% CI)	NA	NA	91·3% (86·4–94·8)	NA	84·7% (78·9–89·4)
	GMC ratio to referent (95% CI)	NA	NA	1·4 (1·1–1·7)	NA	Ref
**1 month after second dose**						
HPV-16						
	n	198	189	190	194	193
	GMC (95% CI)	1507 (1329–1710)	2409 (2116–2742)	3326 (2961–3737)	1352 (1199–1525)	2387 (2091–2725)
	Seropositivity rate (95% CI)	100% (98·2–100)	100% (98·1–100)	100% (98·1–100)	100% (98·1–100)	100% (98·1–100)
	GMC ratio to referent (95% CI)	1·1 (98·3% CI 0·9–1·3)*	1·8 (98·3% CI 1·5–2·2)*	2·4 (98·3% CI 1·9–2·9)*	Ref	1·8 (1·5–2·1)
HPV-18						
	n	200	198	191	197	192
	GMC (95% CI)	383 (338–434)	535 (469–609)	535 (475–603)	306 (269–348)	379 (328–438)
	Seropositivity rate (95% CI)	100% (98·2–100)	100% (98·2–100)	100% (98·1–100)	100% (98·1–100)	100% (98·1–100)
	GMC ratio to referent (95% CI)	1·3 (98·3% CI 1·0–1·5)*	1·7 (98·3% CI 1·4–2·1)*	1·7 (98·3% CI 1·4–2·1)*	Ref	1·2 (1·0–1·5)
**18 months after second dose**						
HPV-16						
	n	198	NA	NA	195	NA
	GMCs (95% CI)	139 (121–159)	NA	NA	119 (104–136)	NA
	Seropositivity rate (95% CI)	100% (98·2–100)	NA	NA	100% (98·1–100)	NA
	GMC ratio to referent (95% CI)	1·1 (0·9–1·3)	NA	NA	Ref	NA
HPV-18						
	n	200	NA	NA	198	NA
	GMC (95% CI)	30 (26–35)	NA	NA	23 (20–27)	NA
	Seropositivity rate (95% CI)	100% (98·2–100)	NA	NA	100% (96·4–99·9)	NA
	GMC ratio to referent (95% CI)	1·3 (1·1–1·6)	NA	NA	Ref	NA

Seropositivity was defined as ≥1·4 IU/mL for HPV-16 and ≥1·1 IU/mL for HPV-18. GMC=geometric mean concentration. HPV=human papillomavirus. IU=international unit. NA=not assessed. 2vHPV=bivalent HPV vaccine. 4vHPV=quadrivalent HPV vaccine.

* Non-inferiority for the primary objectives was declared when the lower bound of the 98·3% CI for the GMC ratio was greater than 0·5.

**Figure 3:**
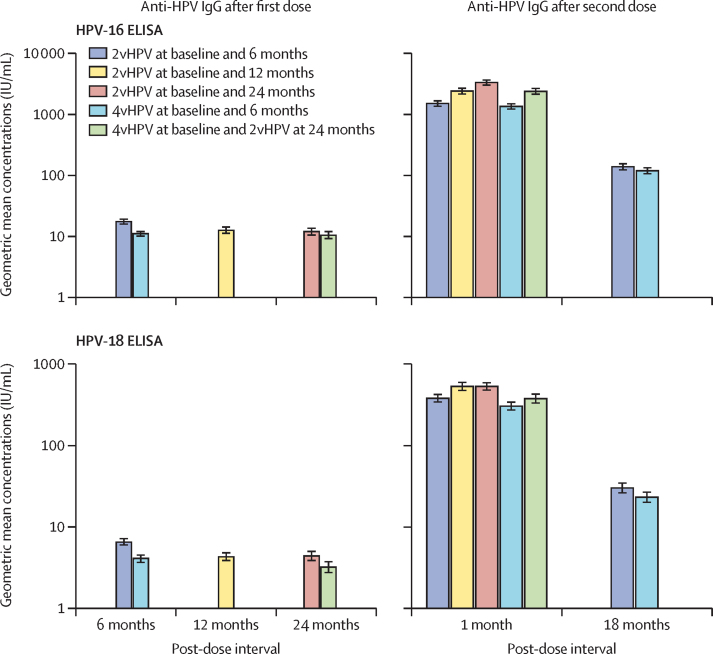
HPV-16 and HPV-18 IgG geometric mean concentrations by ELISA after first and second dose (per-protocol population) Error bars indicate the 95% CIs. HPV=human papillomavirus. IU=international unit. 2vHPV=bivalent HPV vaccine. 4vHPV=quadrivalent HPV vaccine.

Binding antibody concentrations rose sharply following a second dose, reaching a seropositivity rate of 100% for both antigens across all groups. GMCs increased with increasing intervals between the first and second doses of 2vHPV (table 2, figure 3). The mixed-dose schedule was non-inferior to the referent 1 month after the second dose, with a GMC ratio of 1·8 (95% CI 1·5–2·1) for HPV-16 and 1·2 (1·0–1·5) for HPV-18 (table 2). 18 months after the second dose, GMCs decreased similarly in both baseline and 6-month groups, and 2vHPV was non-inferior to 4vHPV, with a GMC ratio of 1·1 (95% CI 0·9–1·3) for HPV-16 and 1·3 (1·1–1·6) for HPV-18 (table 2; appendix 2 p 4). The distribution of GMCs by group and timepoint is in appendix 2 (p 5).

The immunogenicity of a single dose of 2vHPV or 4vHPV was evaluated with samples collected before the second dose in each group. 6, 12, and 24 months following one dose of 2vHPV, the seropositivity rates by ELISA were 98% or higher for HPV-16 at all timepoints and showed a slight decrease from 99% at month 6 to 91% at month 24 for HPV-18. 6 months and 24 months following one dose of 4vHPV, the seropositivity rates were 99% or higher for HPV-16 and showed a slight decrease from 96% at month 6 to 85% at month 24 for HPV-18 (table 2). After a single dose of vaccine, IgG GMCs were lower than after two doses (table 2, figure 3). Following one dose of 2vHPV, HPV-16 and HPV-18 IgG GMCs were highest at 6 months and similar at 12 and 24 months. After a single dose of 4vHPV, HPV-16 and HPV-18 IgG GMCs were similar at 6 and 24 months. 6 months after a single dose of 2vHPV, antibody concentrations were higher than after 4vHPV, whereas 24 months following one dose the 95% CIs of the GMCs overlapped for both antigens (table 2). At 6 months after the first dose, the GMC ratios were 1·5 (95% CI 1·3–1·8) for HPV-16 and 1·6 (1·4–1·9) for HPV-18. At 24 months after the first dose, the GMC ratios were 1·1 (0·9–1·4) for HPV-16 and 1·4 (1·1–1·7) for HPV-18 (table 2; appendix 2 p 4).

HPV-16 and HPV-18 neutralising antibody response, evaluated in a 20% representative sample of participants in the per-protocol population, showed a similar pattern to the ELISA results (appendix 2 p 6). The correlation between PBNA titres and ELISA concentrations was high (*r*≥0·9; appendix 2 p 7).

The frequencies of solicited (overall and local or systemic reactions) and unsolicited adverse events were similar between the study groups (table 3). Pain at the injection site was the most frequently reported local solicited reaction, reported across study groups in 52–67% of participants within the 7 days following vaccination. Local solicited reactions were short lived, with a median duration of 2 days, and mostly mild in intensity, with only one grade 3 report of pain following the first dose of 2vHPV in the group administered 2vHPV at baseline and 6 months. The proportion of participants with local reactions tended to be higher after the second dose (41–51%) than after the first (24–35%; appendix 2 p 8). Solicited systemic reactions within 7 days were reported in 24–37% of participants across groups, with headache, cough, and muscle pain the most frequently reported events. Systemic solicited reactions were short lived, with a median duration of 1–3 days, and mostly mild in intensity, with three events of fever grade 3 or higher in the context of intercurrent infections (one upper respiratory tract infection; two occurrences of malaria). The proportions of participants with systemic reactions were similar after the first and second doses (appendix 2 p 8).

**Table 3:** Safety outcomes (total vaccinated population)

		**2vHPV at baseline and 6 months (N=205)**	**2vHPV at baseline and 12 months (N=206)**	**2vHPV at baseline and 24 months (N=204)**	**4vHPV at baseline and 6 months (N=205)**	**4vHPV at baseline and 2vHPV at 24 months (N=205)**
Any local reaction (solicited within 30 min)		4 (2%, 1–5)	15 (7%, 4–12)	2 (1%, <1–4)	12 (6%, 3–10)	5 (2%, 1–6)
Any local reaction (solicited within 7 days)		131 (64%, 57–70)	113 (55%, 48–62)	106 (52%, 45–59)	138 (67%, 60–74)	120 (59%, 51–65)
	Pain*	131 (64%, 57–70)	113 (55%, 48–62)	106 (52%, 45–59)	138 (67%, 60–74)	119 (58%, 51–65)
Any systemic reaction (solicited within 30 min)		12 (6%, 3–10)	3 (1%, <1–4)	7 (3%, 1–7)	10 (5%, 2–8)	7 (3%, 1–7)
Any systemic reaction (solicited within 7 days)		72 (35%, 29–42)	59 (29%, 23–35)	48 (24%, 18–30)	76 (37%, 30–44)	50 (24%, 19–31)
	Headache*	38 (19%, 14–25)	35 (17%, 12–23)	30 (15%, 10–20)	49 (24%, 18–30)	28 (14%, 9–19)
	Cough*	16 (8%, 5–12)	13 (6%, 3–11)	5 (2%, 1–6)	11 (5%, 3–9)	7 (3%, 1–7)
	Muscle pain*	10 (5%, 2–9)	6 (3%, 1–6)	6 (3%, 1–6)	20 (10%, 6–15)	9 (4%, 2–8)
	Dizziness*	11 (5%, 3–9)	8 (4%, 2–8)	8 (4%, 2–8)	8 (4%, 2–8)	6 (3%, 1–6)
Any non-serious adverse event (unsolicited within 30 days)		43 (21%, 16–27)	41 (20%, 15–26)	46 (23%, 17–29)	52 (25%, 20–32)	34 (17%, 12–22)
	Infections and infestations*	15 (7%, 4–12)	15 (7%, 4–12)	17 (8%, 5–13)	18 (9%, 5–14)	15 (7%, 4–12)
Any serious adverse event (unsolicited for study duration)		4 (2%, 1–5)	0	5 (2%, 1–6)	4 (2%, 1–5)	3 (1%, <1–4)
	Gastrointestinal disorders	1 (<1%, <1–3)	0	0	1 (<1%, <1–3)	0
	General site disorders; administration conditions	1 (<1%, <1–3)	0	0	0	0
	Infections and infestations	2 (1%, <1–4)	0	3 (1%, <1–4)	1 (<1%, <1–3)	2 (1%, <1–4)
	Injury, poisoning, and procedural complications	0	0	2 (1%, <1–4)	1 (<1%, <1–3)	0
	Pregnancy, puerperium, and perinatal conditions	0	0	0	1 (<1%, <1–3)	1 (<1%, <1–3)
Any related serious adverse event (unsolicited for study duration)		0	0	0	0	0
Any adverse event leading to withdrawal (study duration)		0	0	0	0	1 (<1%, <1–3)

Data are n (%, 95% CI), with n as the number of participants with at least one event after either vaccination. HPV=human papillomavirus. 2vHPV=bivalent HPV vaccine. 4vHPV=quadrivalent HPV vaccine.

* Reported in ≥5% of participants.

The reporting rate of non-serious unsolicited adverse events was similar across study groups, ranging from 17% to 25%. All non-serious unsolicited adverse events were considered unrelated to study vaccines and most fell under the infections and infestations System Organ Class. During the study period, 17 serious adverse events in 16 participants were reported; of those, 13 were due to a concurrent illness or condition, three were due to injuries, and one due to a pre‑existing condition (table 3). All serious adverse events resolved except for one case of fatal bacterial sepsis following an unsafe abortion, resulting in study withdrawal. None of the serious adverse events were determined to be related to the study vaccines.

During the study, 14 pregnancies were reported. Two adverse pregnancy outcomes occurred, elective abortion at 34 days and spontaneous abortion at 534 days after the first dose of 4vHPV, and were unrelated to the study vaccines.

## Discussion

In our study, HPV-16 and HPV-18 binding antibody responses in three different two-dose schedules of 2vHPV, regardless of dose interval, were non-inferior to those elicited by the standard two-dose schedule of 4vHPV. Following the second dose, antibody concentrations increased with a larger dosing interval. This observation is consistent with data from other HPV vaccines in girls aged 9–14 years, showing higher antibody titres after a 12-month interval compared with a 6-month interval.^13^ This observation led WHO to suggest an interval of 12 months between doses in a two-dose schedule for programmatic and efficiency reasons, with a minimum 6-month interval. No maximum interval was defined, allowing for longer intervals of up to 3 years or 5 years, should this facilitate immunisation programmes.^4^ The 2vHPV vaccine is licensed in girls younger than 15 years on a two-dose schedule at an interval of 6 months. The data from our study support an extended interval of up to 24 months following the first dose. The study also assessed a mixed-dose schedule (ie, vaccination with products from different manufacturers). Given that use of MSD's 4vHPV vaccine is widespread and WHO prequalification of 2vHPV occurred relatively recently (in 2021), the mixed-dose sequence evaluated in this study was considered the most likely scenario. The mixed-dose schedule elicited at 1 month following the second dose a robust immune response that was non-inferior to the referent group. 1 month following the second dose, the immune response of two doses of 2vHPV administered 24 months apart was higher than that in the mixed-dose schedule, indicating a robust priming with 2vHPV. Little data are available about product interchangeability,^14^ and thus, our data will support policy makers in increasing the flexibility of two-dose HPV vaccine regimens with respect to intervals between doses and mixed-dose schedules.

As described in the literature,^15^, ^16^, ^17^, ^18^ the immune response in our study was approximately 1-log higher 1 month following a second dose than after the first dose; however, the lowest antibody level required for protection is not known. 18 months after the second dose in the groups with a 6-month interval between doses, the antibody concentrations for both antigens had decreased compared with 1 month after the second dose but were higher than those at 6 months after the first dose, with similar concentrations for 2vHPV and 4vHPV groups.

Seropositivity rates by ELISA for both antigens were high 6 months after a single dose of 2vHPV and were more than 91% at 24 months. Similar rates of seropositivity were observed 6 months post-vaccination in a phase 3 study of 2vHPV in girls aged 9–14 years in China.^7^ In our study, seropositivity rates remained high for HPV-16 and HPV-18 6 months following a single dose of 4vHPV vaccine. At 24 months, seropositivity for HPV-16 was high, but seropositivity was lower (85%) for HPV-18. These data are in line with data for 4vHPV from the historical cohort used for immunobridging in the DoRIS study,^19^ for which serology was performed in the same laboratory and with the same assay as for our study. Antibody levels elicited by one dose of 2vHPV were higher at 6 months than at 12 or 24 months, with no difference between the latter two groups. These data suggest that antibody levels plateau 12 months after single-dose vaccination with 2vHPV, with persistence at constant levels at least up to 24 months. The immune response following one dose of 4vHPV was lower than that of 2vHPV at 6 months, but no differences were seen at 24 months. GMCs were lower 6 months after one dose of 2vHPV, and lower for HPV-16 but similar for HPV-18 1 month following the second dose, than observed by Hu and colleagues^7^ in their age-bridging trial, probably due to use of immunogenicity assays based on different virus‑like particle-antigens. Notably, data from the DoRIS study in girls aged 9–14 years in Tanzania 24 months after one dose of 4vHPV^19^ were similar to the data in our study.

The immune response elicited by one dose of 2vHPV compares favourably to that of 4vHPV, for which the efficacy of a single dose was shown in the International Agency for Research on Cancer (IARC) trial in India.^10^ In that trial, a single dose of 4vHPV conferred clinical protection against HPV-16 and HPV-18 infections as high as that of two-dose or three-dose regimens up to 12 years following vaccination.^10^ Applying the principle of immunobridging,^20^ the effectiveness of one dose of 2vHPV can be inferred based on it having similar immunogenicity to 4vHPV for HPV-16 and HPV-18, the two most common oncogenic HPV types. Evidence supporting single-dose efficacy has also been provided in a randomised controlled trial in Kenya that showed the ability of a single-dose regimen of a nonavalent vaccine (MSD, Gardasil9) and a bivalent vaccine (GSK, Cervarix) to prevent persistent HPV-16 and HPV-18 infections in women aged 15–20 years up to 3 years post-vaccination,^21^ in addition to supportive data from the Costa Rica HPV Vaccine trial and the IARC trial in India.^10^, ^15^

In 2022, after reviewing the evidence related to HPV single-dose vaccination, WHO updated their position paper for HPV vaccination^4^ to include a one-dose regimen in girls and women aged 9–20 years as an alternate option to two doses. Of note, this recommendation does not apply to people who are immunocompromised, including those living with HIV, who should receive two or preferably three doses of HPV vaccines. As of November, 2024, 61 countries have either initiated or switched to single-dose HPV vaccination.^22^ WHO limits its recommendation to vaccines for which single-dose use is supported by efficacy data or immunobridging studies, wherein a single dose must have peak and 24-month plateau antibody levels that are comparable to those of vaccines with proven single-dose efficacy.^4^ We found that antibody levels following one dose of 2vHPV were similar at 12 and 24 months, suggesting that 24 months is well within the plateau phase. Our study has shown that antibody levels at both 6 and 24 months following one dose of 2vHPV were similar to those of 4vHPV, a vaccine with demonstrated single-dose efficacy, at each timepoint. Consequently, our data support the consideration of Innovax's 2vHPV vaccine for single-dose use and were the basis for its inclusion among vaccines recommended for use in a single-dose schedule by WHO.^5^

IgG-binding antibody, measured by ELISA, a high-throughput assay, was selected as the primary endpoint to evaluate immunogenicity. The neutralising immune response, thought to be the mechanism of protection for prophylactic HPV vaccines,^23^ was evaluated in a subset of participants and showed a similar trend to the responses observed in the binding assay. We were also able to show a good correlation between both assays in a wide variety of antibody concentrations, which is consistent with the literature.^24^

Both study vaccines were well tolerated and showed similar safety profiles. Solicited and unsolicited adverse events occurred at similar frequencies between study groups. Serious adverse events were rare, were determined to be unrelated to vaccination, and occurred in the context of concurrent illnesses or conditions, due to injury or a pre-existing condition.

Strengths of our study include the evaluation of 2vHPV in two distinct LMIC settings (and the first assessment of the vaccine outside the country of licensure), the evaluation of several vaccination schedules, and the generation of novel one-dose data for Innovax's 2vHPV vaccine in a robust number of participants. There was high compliance with the protocol and excellent retention during the study. The immunogenicity assessments used validated assays with high reproducibility that were based on product-agnostic antigens, thus allowing for proper immunobridging evaluations not specifically linked to either of the study vaccines. The immune response with a high-throughput binding assay was complemented by evaluation with a neutralising assay.

Limitations of this study include a healthy study population, excluding people with immunocompromising conditions, and its open-label design. However, the primary immunological objectives were assessed in a blinded manner, thereby minimising the potential for bias. In alignment with other studies,^7^, ^12^, ^16^, ^19^, ^25^ we assessed non-inferiority using a margin for the lower bound of the CI for the GMC ratio of greater than 0·5; however, the non-inferiority comparisons in our study would have met a threshold of 0·67 (note the selected non-inferiority limit affects the sample size calculation, but not the study outcomes). Another limitation is that the evaluation of the one-dose regimen was an exploratory objective and did not include a longitudinal assessment of antibody concentrations over time but rather evaluation of different cohorts of participants at each timepoint. However, the sample size was robust, the groups had similar baseline characteristics, and a similar method was applied to sample collection across the groups. Furthermore, all participants were required per protocol to receive a second dose, and thus an evaluation of antibody persistence longer than 2 years following one dose of 2vHPV will require subsequent research. Nevertheless, the immunogenicity pattern in our study suggests that persistence following single-dose vaccination will be long lasting, similar to what is seen for other HPV vaccines.^15^, ^18^ Finally, to keep the study at a manageable size, a mixed-dose schedule of 2vHPV followed by 4vHPV was not included, and the referent was limited to two doses of 4vHPV given 6 months apart.

In conclusion, the immunogenicity of Innovax's 2vHPV vaccine administered in a two-dose schedule 6, 12, or 24 months apart was non-inferior to that of two doses of MSD's 4vHPV vaccine administered 6 months apart. Immunogenicity increased with longer intervals between dosing, and a mixed-dose schedule was highly immunogenic, supporting a flexible dosing schedule and vaccine interchangeability. Immunogenicity following one dose of 2vHPV was similar to that following one dose of 4vHPV at 6 and 24 months. Because single-dose efficacy has been established for the referent vaccine, the similar immunogenicity across the two vaccines supports considering 2vHPV for single-dose use. Both vaccines were safe and well tolerated. Innovax's bivalent vaccine presents an affordable alternative for HPV vaccination on a one-dose or two-dose schedule and should increase access to safe and efficacious HPV vaccines in LMICs, which will contribute to cervical cancer elimination efforts.

### Contributors

### Data sharing

De-identified data that underlie the results in this report will be made available to others in the scientific community upon request. Standard criteria for making data available for valid research projects will be used following application by suitably qualified researchers. For data access, please contact cviadatarequests@path.org.

## Declaration of interests

We declare no competing interests.

## Supplementary Materials

Bengali translation of the abstract
