# Supplementary material for: Immunogenicity and safety of an Escherichia coli-produced bivalent human papillomavirus vaccine (Cecolin) in girls aged 9–14 years in Ghana and Bangladesh: a randomised, controlled, open-label, non-inferiority, phase 3 trial
Source: Lancet Infect Dis. 2025 Aug;25(8):861–72. doi: 10.1016/S1473-3099(25)00031-3 (PMC12287245; doi:10.1016/S1473-3099(25)00031-3)
Supplement: Bengali translation of the abstract [file mmc1.pdf]

THE LANCET
Infectious Diseases

## Supplementary appendix 1

This translation in Bangla was submitted by the authors and we reproduce it as supplied. It has not been peer reviewed. *The Lancet*'s editorial processes have only been applied to the original in English, which should serve as reference for this manuscript.

'এই [বাংলায়] অনুবাদটি লেখকরা জমা দিয়েছিলেন এবং এটি যেমনভাবে দেওয়া হয়েছে আমরা সেইভাবেই পুনরায় বর্ণনা করছি। এটি কোনো সমকক্ষ ব্যক্তি পর্যালোচনা করেননি। দ্য ল্যানসেট-এর সম্পাদকীয় প্রক্রিয়াগুলি শুধুমাত্র মূল ইংরেজিতে প্রয়োগ করা হয়েছে, যা এই পাণ্ডুলিপির রেফারেন্স হিসাবে কাজ করবে।'

Supplement to: Agbenyega T, Schuind AE, Adjei S, et al. Immunogenicity and safety of an *Escherichia coli*-produced bivalent human papillomavirus vaccine (Cecolin) in girls aged 9–14 years in Ghana and Bangladesh: a randomised, controlled, open-label, non-inferiority, phase 3 trial. *Lancet Infect Dis* 2025; published online March 19. https://doi.org/10.1016/S1473-3099(25)00031-3.

## সারাংশ

**পটভূমিঃ** এইচপিভি টিকা প্রায় ২০ বছর ধরে পাওয়া যাচ্ছে। তবে, ১৫ বছর বা তার কম বয়সী মেয়েদের সামগ্রিক আওতাভুক্তি কম, বিশেষ করে কম সম্পদশীল স্থান যেখানে জরায়ুমুখের ক্যান্সারের ঝুঁকি সবচেয়ে বেশি। এইচপিভি টিকাদানের প্রবেশাধিকার বৃদ্ধি এবং বাস্তবায়ন সহজতর করা জরায়ুমুখের ক্যান্সার নির্মূল প্রচেষ্টায় অবদান রাখবে। বিভিন্ন ডোজিং পদ্ধতিতে এবং কম-সম্পদশীল জায়গায় ফলাফলের জন্য, আমরা বহুল ব্যবহৃত কোয়াড্রিভ্যালেন্ট ভ্যাকসিনের তুলনায় *E. coli* দ্বারা প্রকাশিত বাইভ্যালেন্ট হিউম্যান প্যাপিলোমাভাইরাস (HPV) ভ্যাকসিনের (2vHPV) বিভিন্ন সময়সূচীর নিরাপত্তা এবং রোগ প্রতিরোধ ক্ষমতা মূল্যায়নের লক্ষ্য রেখেছিলাম।

**পদ্ধতিঃ** এই দৈবচয়ন পদ্ধতিতে নিয়ন্ত্রিত, উন্মুক্ত, অনিম্নমানতা, তৃতীয় ধাপের ট্রায়ালে ঘানা এবং বাংলাদেশের একক স্থান থেকে ৯-১৪ বছর বয়সী সুস্থ মেয়েদের অর্ন্তভুক্তি করা হয়েছিল। অংশগ্রহণকারীদের ইন্টারেক্টিভ ওয়েব রেসপন্স সিস্টেম প্রযুক্তির মাধ্যমে স্থান অনুসারে পাঁচটি গবেষণা গোষ্ঠীতে সমানভাবে ভাগ করা হয়েছিল: 2vHPV এর দুটি ডোজ, একটি বেসলাইনে এবং একটি ৬, ১২, অথবা ২৪ মাস পরে; কোয়াড্রিভ্যালেন্ট ভ্যাকসিনের (4vHPV) একটি ডোজ বেসলাইনে এবং ২৪ মাস পরে 2vHPV একটি ডোজ; অথবা ৬ মাসের ব্যবধানে 4vHPV এর দুটি ডোজ দেওয়া হয়েছিল (তুলনাকারী)। আমরা বেসলাইনে এবং দ্বিতীয় ডোজের আগে এবং ১ মাস পরে ELISA দ্বারা অ্যান্টিজেন-নির্দিষ্ট (HPV-16 এবং HPV-18) বাইন্ডিং অ্যান্টিবডিগুলির জন্য পরীক্ষা করেছি। প্রাথমিক উদ্দেশ্য ছিল জিওমেট্রিক মিন কনসেন্ট্রেশন (GMC) অনুপাতের জন্য ৯৮.৩% কনফিডেন্স ইন্টারভাল (CI) এর নিম্নসীমার ০.৫ মার্জিন সহ গবেষণায় অংশগ্রহণকারীদের ডোজ ২ এর এক মাস পরে তুলনাকারী টীকার সময়সূচীর সাথে বাইভ্যালেন্ট টীকার (2vHPV) রোগ-প্রতিরোধক অনিম্নমানতা প্রদর্শন করা । মোট টিকাপ্রাপ্ত জনসংখ্যার ক্ষেত্রে বিরূপ প্রতিক্রিয়া এবং গুরুতর বিরূপ প্রতিক্রিয়াগুলিকে মূল্যায়ন করা হয়েছিল। গবেষণাটি ClinicalTrials.gov NCT No. 04508309 এ নিবন্ধিত ছিল এবং সম্পন্ন হয়েছে।

**ফলাফলঃ** ১৫ মার্চ, ২০২১ থেকে ১৮ নভেম্বর, ২০২১ এর মধ্যে, ১০২৫ জন মেয়েকে অন্তর্ভুক্ত করা হয়েছিল যাতে করে 2vHPV টিকা বেসলাইনে এবং ৬ মাস (n=২০৫), ১২ মাস (n=২০৬), অথবা ২৪ মাস (n=২০৪); 4vHPV টিকা বেসলাইনে এবং ৬ মাস (n=২০৫); অথবা বেসলাইনে 4vHPV এবং ২৪ মাস 2vHPV (n=২০৫) পেয়েছিল । পার-প্রোটোকল বিশ্লেষণে বিভিন্ন গ্রুপের ৯৬-৯৯% অংশগ্রহণকারীদের অন্তর্ভুক্ত করা হয়েছিল। দ্বিতীয় ডোজ দেওয়ার এক মাস পরে, 2vHPV অনিম্নমানতা দেখানো হয়েছিল, HPV-16 এর জন্য GMC অনুপাত ১.১ এবং ২.৪ (০.৯ এবং ১.৯ এর মধ্যে GMC অনুপাতের ৯৮.৩% CI এর নিম্ন সীমা) এবং HPV-18 এর জন্য ১.৩ এবং ১.৭ (১.০ এবং ১.৪) এর মধ্যে ছিল। একটি অনুসন্ধানমূলক উদ্দেশ্য হিসাবে, আমরা 2vHPV টিকার এক ডোজ পরে ইমিউনোজেনিসিটি মূল্যায়ন করেছি, দেখেছি যে এটি ২৪ মাস পর্যন্ত 4vHPV এর অনুরূপ ছিল, HPV-16 এর জন্য ২৪ মাসের ১.১ (৯৫% CI ০.৯ - ১.৪) এবং HPV-18 এর জন্য ১.৪ (১.১ - ১.৭) GMC অনুপাত সহ । বিভিন্ন গবেষণা গোষ্ঠীতে প্রতিকূল ঘটনার ফ্রিকোয়েন্সি একই রকম ছিল, কোনও অযাচিত ঘটনা রিপোর্ট করা হয়নি। SAE গুলি বিরল ছিল এবং কোনটিই টিকাদানের সাথে সম্পর্কিত বলে নির্ধারিত হয়নি।

**ব্যাখ্যাঃ** 2vHPV এর বর্ধিত দুই-ডোজ টীকার ইমিউনোজেনিসিটি ডোজিং পরিবর্তনযোগ্যতাকে সমর্থন করে। 2vHPV এর এক ডোজ 4vHPV এর অনুরূপ ইমিউনোজেনিসিটি প্রদর্শন করেছে, যার জন্য একক ডোজ কার্যকারিতা ২৪ মাস পর্যন্ত প্রদর্শিত হয়েছে যা একক ডোজ ব্যবহারকে সমর্থন করে।



**অর্থায়নঃ** বিল অ্যান্ড মেলিন্ডা গেটস ফাউন্ডেশন এবং জার্মান ফেডারেল শিক্ষা ও গবেষণা মন্ত্রণালয় এবং ইমিউনোলজিক্যাল পরীক্ষার আংশিক অর্থায়ন করেছিল জাতীয় ক্যান্সার ইনস্টিটিউট, জাতীয় স্বাস্থ্য ইনস্টিটিউট।



কপিরাইট © ২০২৫ লেখক(রা)। এলসেভিয়ার লিমিটেড দ্বারা প্রকাশিত। এটি CC By ৪.০ লাইসেন্সের অধীনে একটি ওপেন অ্যাক্সেস নিবন্ধন।
