# Supplementary material for: Immunogenicity and safety of an Escherichia coli-produced bivalent human papillomavirus vaccine (Cecolin) in girls aged 9–14 years in Ghana and Bangladesh: a randomised, controlled, open-label, non-inferiority, phase 3 trial
Source: Lancet Infect Dis. 2025 Aug;25(8):861–72. doi: 10.1016/S1473-3099(25)00031-3 (PMC12287245; doi:10.1016/S1473-3099(25)00031-3)
Supplement: Supplementary appendix 2 [file mmc2.pdf]

THE LANCET
Infectious Diseases

Supplementary appendix 2

This appendix formed part of the original submission and has been peer reviewed. We post it as supplied by the authors.

Supplement to: Agbenyega T, Schuind AE, Adjei S, et al. Immunogenicity and safety of an *Escherichia coli*-produced bivalent human papillomavirus vaccine (Cecolin) in girls aged 9–14 years in Ghana and Bangladesh: a randomised, controlled, open-label, non-inferiority, phase 3 trial. *Lancet Infect Dis* 2025; published online March 19. https://doi.org/10.1016/S1473-3099(25)00031-3.

Supplementary Appendix 2

Immunogenicity and safety of an Escherichia coli-produced bivalent human papillomavirus vaccine (Cecolin) in girls 9–14 years of age in Ghana and Bangladesh: a randomized, controlled, open-label, non-inferiority, phase 3 trial

Authors

Tsiri Agbenyega, Anne E. Schuind, Samuel Adjei, Kalpana Antony, John J Aponte, Patrick BY Buabeng et al.

Table of Contents

Supplementary Methods 2

Clinical Assays 2

Figure S1 – Reasons for exclusion from per protocol population for immunogenicity by HPV-16 and HPV-18 ELISA assay 3

Figure S2 - HPV-16 and HPV-18 IgG geometric mean concentrations ratios by ELISA post Dose 1 and post Dose 2 (per protocol population) 4

Figure S3 - HPV-16 and HPV-18 IgG antibody concentrations by ELISA (per protocol population) 5

Table S1 - HPV-16 and HPV-18 neutralizing responses by PBNA (per protocol population) 6

Figure S4 - Correlation of HPV-16 and HPV-18 binding antibodies (ELISA) and neutralizing antibodies (PBNA) 2vHPV (total vaccinated population) 7

Table S2 - Solicited adverse events reported at a frequency >2% in any group post Dose 1 and Dose 2 (total vaccinated population) 8

References 9

Supplementary Methods

Clinical Assays

Immunologic testing was performed by the Frederick National Laboratory for Cancer Research in Frederick, Maryland, United States. It included measurement of antigen specific (HPV-16 or HPV-18) binding antibodies, as primary assay and, in a representative subset of 20% of participants, neutralizing antibodies by pseudovirion-based neutralization assay (PBNA).

Anti-HPV16 and anti-HPV18 immunoglobulin G (IgG) and neutralizing antibodies were previously described[1]. The antibody binding HPV16 and HPV18 VLP-based ELISA was performed by coating polystyrene flat-bottom microtiter plates (MaxiSorp, high binding; Nunc, Thermo Fisher Scientific, USA) with HPV16 or HPV18 VLPs. Prior to use, the plates are washed and blocked with 4% skim milk and 0.2% Tween 20 in phosphate-buffered saline. Following incubation, the plates are washed, and participant's serum are initially diluted 1/100 and serially diluted in two-fold increments in the assay plate to a final dilution of 1/12,800. The plates are incubated for one hour at room temperature. After washing the plate, peroxidase-labeled goat anti-human IgG (KPL, Inc., Gaithersburg, MD) was added for one hour at room temperature. After washing the plate, the plate was then developed with a tetramethylbenzidine (TMB) substrate solution (KPL, Inc.) for 25 minutes in the dark at room temperature. Next, the reaction was stopped with 0.36 N sulfuric acid, and the absorbance (450 nm – 620 nm) measured with a plate reader (Spectramax; Molecular Devices, San Jose, CA). Antibody levels, expressed as International Units (IU)/mL, are calculated by interpolation of OD values from the standard curve by averaging the calculated concentrations from all dilutions that fall within the working range of the standard curve. The lower limit of quantitation of the HPV16 assay was 1.4 IU/mL, and the lower limit of quantitation of the HPV18 assay was 1.1 IU/mL.

Neutralizing antibody responses were measured utilizing pseudovirions (PSV) with a secreted alkaline phosphatase reporter gene and 293TT cells. First, 293TT cells are seeded in a 96-well, flat- bottom plate at a cell density of $3x10^4$ cells per well and incubated at 37ºC and 5% CO_2 for 2 or more hours before addition of controls and diluted samples. Twenty-five microliters of serially diluted participant serum (diluted 1/10 in 4-fold increments up to 1/163,840) are incubated with 100 µL of HPV16 or HPV18 (PsV) in duplicate at 4ºC for 1 hour. Following 4ºC incubation, the samples are transferred to the 293TT cells at 37ºC for 72 hours. Following 37ºC incubation, the clarified supernatants are transferred to 96-well plates and frozen at <-10ºC until further testing. The Great EscAPe Secreted Alkaline Phosphatase (SEAP) assay kit was used according to the manufacturer's protocol (Takara-Clontech Laboratories, Inc.). Neutralization titers are calculated by linear interpolation and defined as the reciprocal of the dilution that caused 50% reduction in SEAP activity compared with control wells. The reported neutralization titers reflect the mean value of duplicate testing for each sample. The lower limit of quantitation of the HPV16 and HPV18 PsV assay was a titer of <21 and <16, respectively.

The assays were controlled during testing through several methods: use of the same lot of VLPs, internal reference reagent (standard), and controls (seropositive and seronegative) throughout all testing. Furthermore, controls and standard were included on every plate to ensure each plate passed stringent quality control criteria. Lastly, the longitudinal collections of each subject were tested within the same batch and subjects from both collection sites were included into the same batch as well to minimize batch-to-batch effects across study sites.

Figure S1 – Reasons for exclusion from per protocol population for immunogenicity by HPV-16 and HPV-18 ELISA assay

2vHPV at baseline and 6 months

Primary Endpoint
1-month post-dose 2

Total Vaccinated Population
(**n=205**)

Per-Protocol Population
Included in anti-HPV 16 and/or anti-HPV 18 PPP (**n=204**)

Anti-HPV 16
Included **(n=198)**
Excluded (n=7)*

- Baseline seropositive (n=6)
- Missing baseline result (n=1)

Anti-HPV 18
Included **(n=200)**
Excluded (n=5)*

- Baseline seropositive (n=5)

2vHPV at baseline and 12 months

Primary Endpoint
1-month post-dose 2

Total Vaccinated Population
(n=206)

Per-Protocol Population
Included in anti-HPV 16 and/or anti-HPV 18 PPP (**n=200**)

Anti-HPV 16
Included **(n=189)**
Excluded (n=17)*

- Baseline seropositive (n=15)
- Missed dose 2 (n=2)
- Missing result (n=2)
- Randomization error (n=1)

Anti-HPV 18
Included **(n=198)**
Excluded (n=8)*

- Baseline seropositive (n=5)
- Missed dose 2 (n=2)
- Missing result (n=2)
- Randomization error (n=1)

2vHPV at baseline and 24 months

Primary Endpoint
1-month post-dose 2

Total Vaccinated Population
(n=204)

Per-Protocol Population
Included in anti-HPV 16 and/or anti-HPV 18 PPP (**n=196**)

Anti-HPV 16
Included **(n=190)**
Excluded (n=14)*

- Baseline seropositive (n=9)
- Missed dose 2 (n=5)
- Missed visit (n=4)
- Met exclusion criterion (n=1)
- Early termination (n=1)

Anti-HPV 18
Included **(n=191)**
Excluded (n=13)*

- Baseline seropositive (n=7)
- Missed dose 2 (n=5)
- Missed visit (n=4)
- Met exclusion criterion (n=1)
- Early termination (n=1)

4vHPV at baseline and 6 months (Control)

Primary Endpoint
1-month post-dose 2

Total Vaccinated Population
(n=205)

Per-Protocol Population
Included in anti-HPV 16 and/or anti-HPV 18 PPP (**n=201**)

Anti-HPV 16
Included **(n=194)**
Excluded (n=11)*

- Baseline seropositive (n=8)
- Early termination (n=1)
- Missing baseline result (n=1)
- Sample out of window (n=1)

Anti-HPV 18
Included **(n=197)**
Excluded (n=8)*

- Baseline seropositive (n=6)
- Early termination (n=1)
- Sample out of window (n=1)

4vHPV vaccine at baseline and 2vHPV at 24 months

Secondary Endpoint
1-month post-dose 2

Total Vaccinated Population
(n=205)

Per-Protocol Population
Included in anti-HPV 16 and/or anti-HPV 18 PPP (**n=198**)

Anti-HPV 16
Included **(n=193)**
Excluded (n=12)*

- Baseline seropositive (n=7)
- Missed dose 2 (n=4)
- Missing result (n=4)
- Early termination (n=1)

Anti-HPV 18
Included **(n=192)**
Excluded (n=13)*

- Baseline seropositive (n=8)
- Missed dose 2 (n=4)
- Missing result (n=4)
- Early termination (n=1)

* Participants could be excluded for multiple reasons
PPP = Per Protocol Population; HPV = human papillomavirus; 2vHPV = bivalent HPV vaccine; 4vHPV = quadrivalent HPV vaccine

Figure S2 - HPV-16 and HPV-18 IgG geometric mean concentrations ratios by ELISA post Dose 1 and post Dose 2 (per protocol population)

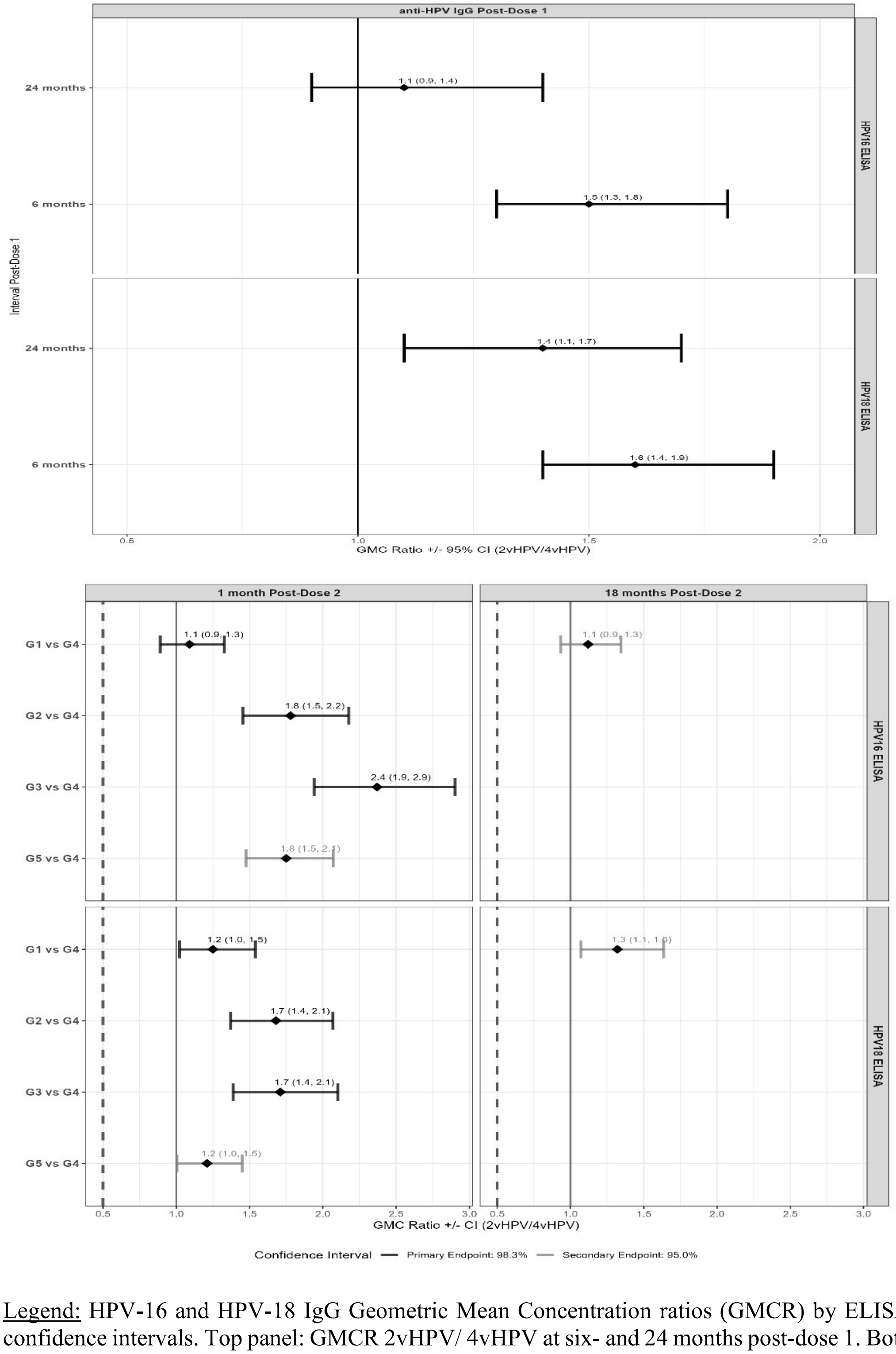

Legend: HPV-16 and HPV-18 IgG Geometric Mean Concentration ratios (GMCR) by ELISA and associated 95% confidence intervals. Top panel: GMCR 2vHPV/ 4vHPV at six- and 24 months post-dose 1. Bottom panel: co-primary (black) and secondary (grey) GMCR (2vHPV/ 4vHPV) by study group at one month and 18 months post-Dose 2. Non-inferiority established for co-primary and secondary endpoints if lower bound of the confidence interval is greater than 0·5. G1: 2vHPV at baseline and 6 months; G2: 2vHPV at baseline and 12 months; G3: 2vHPV at baseline and 24 months; G4: 4vHPV at baseline and 6 months (referent); G5: 4vHPV at baseline and 2vHPV at 24 months.

Figure S3 - HPV-16 and HPV-18 IgG antibody concentrations by ELISA (per protocol population)

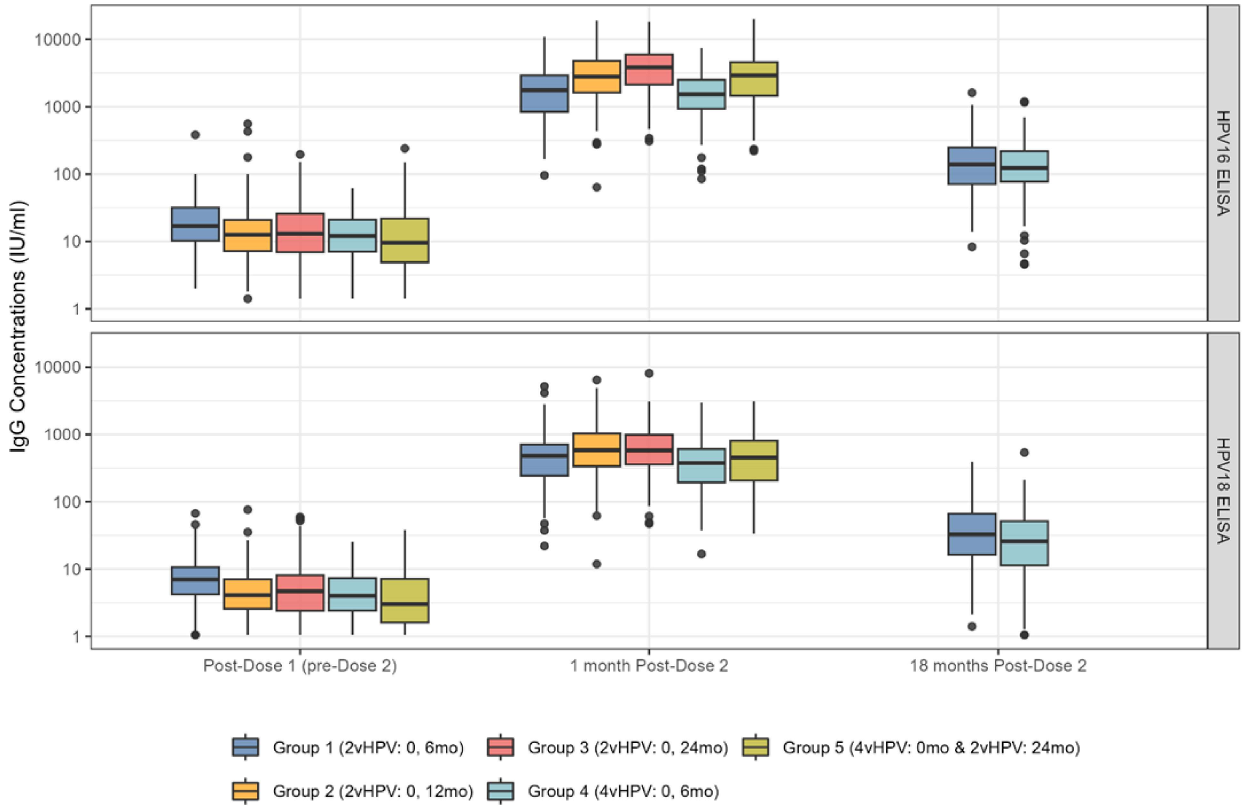

Legend: Boxplot of HPV-16 and HPV-18 IgG concentrations (IU/mL) by enzyme-linked immunosorbent assay (ELISA) by study group and timepoint showing median, interquartile range, and minimum/ maximum values.

Table S1 - HPV-16 and HPV-18 neutralizing responses by PBNA (per protocol population)

	2vHPV at baseline and 6 months	2vHPV at baseline and 12 months	2vHPV at baseline and 24 months	4vHPV at baseline and 6 months (Control)	4vHPV vaccine at baseline and 2vHPV at 24 months
**Neutralizing antibody response (PBNA) post Dose 1**					
	6 mo. post Dose 1 2vHPV	12 mo. Post Dose 1 2vHPV D1	24 mo. Post Dose 1 2vHPV	6 mo. Post Dose 1 4vHPV	24 mo. Post Dose 1 4vHPV
**HPV-16**					
**N**	40	41	41	40	40
**GMT (95% CI)**	104 (79; 138)	95 (73; 123)	126 (95; 166)	72 (54; 96)	125 (85; 185)
**Seropositivity rate (95% CI)**	97·5% (86·8; 99·9)	100% (91·4; 100)	97·6% (87·1; 99·9)	90% (76·3; 97·2)	95% (83·1; 99·4)
**HPV-18**					
**N**	40	41	40	41	41
**GMT (95% CI)**	66 (42; 103)	51 (41; 64)	78 (58; 105)	48 (39; 68)	44 (35; 95)
**Seropositivity rate (95% CI)**	92·5% (79·6; 98·4)	90·2% (76·9; 97·3)	95% (83·1; 99·4)	97·6% (87·1; 99·9)	80·5% (65·1; 91·2)
**Antibody response (PBNA) one month post Dose 2**					
**HPV-16**					
**N**	40	41	40	40	38
**GMT (95% CI)**	16791 (11641; 24219)	32020 (25011; 40993)	48615 (36061; 65541)	14281 (10532; 19366)	31459 (24295; 40736)
**Seropositivity rate (95% CI)**	100% (91·2; 100)	100% (91·4; 100)	100% (91·2; 100)	100% (91·2; 100)	100% (90·7; 100)
**HPV-18**					
**N**	40	41	39	41	39
**GMT (95% CI)**	6082 (3548; 10425)	9304 (7111; 12173)	11205 (8351; 15035)	6774 (5045; 9098)	7549 (5474; 10411)
**Seropositivity rate (95% CI)**	97·5% (86·8; 99·9)	100% (91·4; 100)	100% (91·0; 100)	100% (91·4; 100)	100% (91·0; 100)
**Neutralizing antibody response (PBNA) 18 months post Dose 2**					
**HPV-16**					
**N**	40	··	··	40	··
**GMT (95% CI)**	1542 (1077; 2208)	··	··	1368 (999; 1874)	··
**Seropositivity rate (95% CI)**	100% (91·2; 100)	··	··	100% (91·2; 100)	··
**HPV-18**					
**N**	40	··	··	41	··
**GMT (95% CI)**	442 (302; 647)	··	··	423 (290; 619)	··
**Seropositivity rate (95% CI)**	100% (91·2; 100)	··	··	97·6% (87·1; 99·9)	··

PBNA: pseudovirion-based neutralization assay. Seropositivity rate (antibody titers ≥21 for HPV-16 and ≥16 for HPV-18); GMTs: Geometric Mean Titers; CI: Confidence Interval; mo = months; N: number of subjects in PP; PP: Per protocol population (subjects seronegative at baseline)

Figure S4 - Correlation of HPV-16 and HPV-18 binding antibodies (ELISA) and neutralizing antibodies (PBNA) 2vHPV (total vaccinated population)

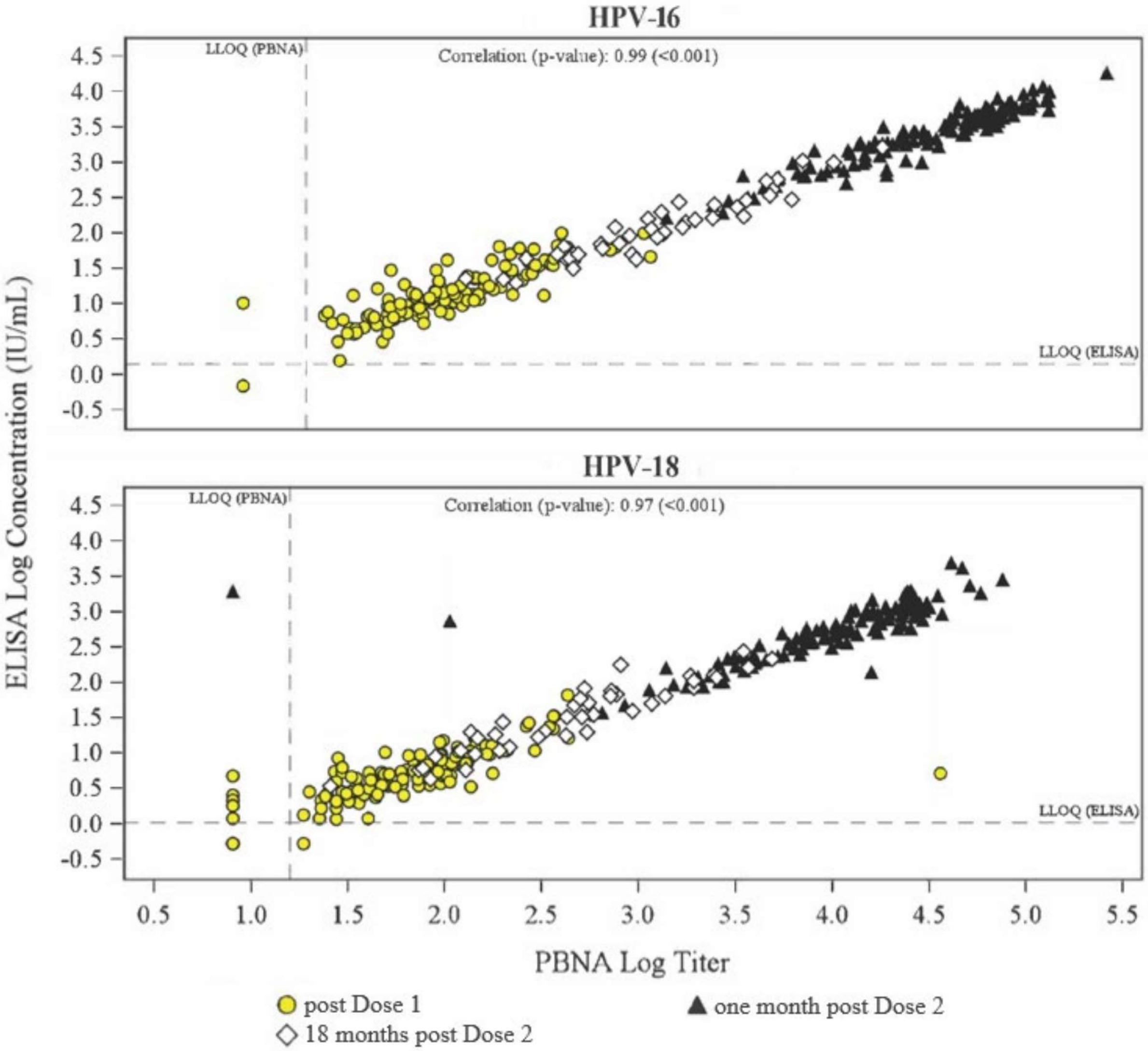

Table S2 - Solicited adverse events reported at a frequency >2% in any group post Dose 1 and Dose 2 (total vaccinated population)

	2vHPV at baseline and 6 months	2vHPV at baseline and 12 months	2vHPV at baseline and 24 months	4vHPV at baseline and 6 months (Control)	4vHPV at baseline and 2vHPV at 24 months
Safety endpoints	n (%)	n (%)	n (%)	n (%)	n (%)
	95% CI	95% CI	95% CI	95% CI	95% CI
**Post Dose 1**					
**N**	**205**	**206**	**204**	**205**	**205**
Any local reaction (solicited/ 7 days)	64 (31)	49 (24)	48 (24)	72 (35)	72 (35)
	25; 38	18; 30	18; 30	29; 42	29; 42
Pain	64 (31)	49 (24)	48 (24)	72 (35)	72 (35)
	25; 38	18; 30	18; 30	29; 42	29; 42
Any systemic reaction (solicited/ 7 days)	32 (16)	35 (17)	32 (16)	41 (20)	33 (16)
	11; 21	12; 23	11; 21	15; 26	11; 22
Headache	25 (12)	20 (10)	20 (10)	30 (15)	19 (9)
	8; 17	6; 15	6; 15	10; 20	6; 14
Cough	2 (1)	5 (2)	3 (1·5)	5 (2)	7 (3)
	0·1; 3	1; 6	0·3; 4	1; 6	1; 7
Muscle pain	4 (2)	4 (2)	4 (2)	9 (4)	7 (3)
	0·5; 5	0·5; 5	0·5; 5	2; 8	1; 7
Fatigue	1 (0·5)	1 (0·5)	5 (3)	3 (2)	2 (1)
	0·01; 3	0·01; 3	1; 6	0·3; 4	0·1; 3
Dizziness	3 (2)	6 (3)	4 (2)	4 (2)	3 (2)
	0·3; 4	1; 6	1; 5	1; 5	0·3; 4
Fever	3 (2)	0 (0)	2 (1)	6 (3)	1 (0·5)
	0·3; 4		0·1; 4	1; 6	0·01; 3
Vomiting	4 (2)	1 (0·5)	2 (1)	7 (3)	1 (0·5)
	1; 5	0·01; 3	0·1; 4	1; 7	0·01; 3
Nausea	5 (2)	3 (2)	4 (2)	4 (2)	3 (2)
	1; 6	0·3; 4	1; 5	1; 5	0·3; 4
Chills	2 (1)	2 (1)	2 (1)	5 (2)	0 (0)
	0·1; 3	0·1; 3	0·1; 4	1; 6	
**Post Dose 2**					
**N**	**205**	**204**	**199**	**205**	**201**
Any local reaction (solicited/ 7 days)	101 (49)	92 (45)	82 (41)	104 (51)	87 (43)
	42; 56	38; 52	34; 48	44; 58	36; 50
Pain	101 (49)	92 (45)	82 (41)	104 (51)	86 (43)
	42; 56	38; 52	34; 48	44; 58	36; 50
Any systemic reaction (solicited/ 7 days)	49 (24)	34 (17)	23 (12)	47 (23)	24 (12)
	18; 30	12; 23	7; 17	17; 29	8; 17
Headache	20 (10)	20 (10)	14 (7)	27 (13)	13 (7)
	6; 15	6; 15	4; 12	9; 19	3; 11
Cough	14 (7)	8 (4)	2 (1)	6 (3)	1 (0·5)
	4; 11	2; 8	0·1; 4	1; 6	0·01; 3
Muscle pain	6 (3)	2 (1)	2 (1)	11 (5)	3 (2)
	1; 6	0·1; 4	0·1; 4	3; 9	0·3; 4
Fatigue	4 (2)	1 (0·5)	5 (3)	2 (1)	4 (2)
	0·5; 5	0·01; 3	1; 6	0·1; 3	0·5; 5
Dizziness	8 (4)	3 (2)	4 (2)	4 (2)	3 (2)
	2; 8	0·3; 4	1; 5	1; 5	0·3; 4

Fever: temperature ≥ 38° Celsius; N = number of subjects having received Dose 1 or Dose 2. n = number of subjects with at least one event after Dose 1 or Dose 2.

References

1. Zaman K, Schuind AE, Adjei S, et al. Safety and immunogenicity of Innovax bivalent human papillomavirus vaccine in girls 9-14 years of age: Interim analysis from a phase 3 clinical trial. Vaccine 2024; **42**: 2290–8.
